# *Staphylococcus aureus* Tetracycline Resistance and Co-resistance in a Doxycycline Postexposure Prophylaxis–Eligible Population

**DOI:** 10.1093/infdis/jiae634

**Published:** 2024-12-24

**Authors:** Rachel Mittelstaedt, Sanjat Kanjilal, David Helekal, Gregory K Robbins, Yonatan H Grad

**Affiliations:** Division of Infectious Diseases, Department of Medicine, Massachusetts General Hospital; Department of Immunology and Infectious Diseases, Harvard T. H. Chan School of Public Health; Department of Population Medicine, Harvard Medical School and Harvard Pilgrim Healthcare Institute; Division of Infectious Diseases, Brigham and Women's Hospital, Boston, Massachusetts; Department of Immunology and Infectious Diseases, Harvard T. H. Chan School of Public Health; Division of Infectious Diseases, Department of Medicine, Massachusetts General Hospital; Department of Immunology and Infectious Diseases, Harvard T. H. Chan School of Public Health

**Keywords:** doxycycline postexposure prophylaxis, doxy-PEP, *Staphylococcus aureus*

## Abstract

Among doxycycline postexposure prophylaxis (doxy-PEP)–eligible men, *Staphylococcus aureus* tetracycline nonsusceptibility is more prevalent than in the overall population and is associated with resistance to trimethoprim-sulfamethoxazole and clindamycin. Doxy-PEP may select for multidrug-resistant *S aureus*, underscoring the importance of surveillance.

Rates of bacterial sexually transmitted infections (STIs) have been rising in the United States (US) over much of the past decade [1]. Several randomized controlled trials demonstrated that doxycycline postexposure prophylaxis (doxy-PEP), prescribed as 200 mg of doxycycline taken within 72 hours of condomless sex, reduces the incidence of bacterial STIs among men who have sex with men (MSM) and transgender women (TGW) [2]. These findings prompted national guidelines recommending consideration of doxy-PEP for MSM and TGW, particularly those who have had a bacterial STI in the past 12 months [3].

One concern with doxy-PEP is that it may select for resistance to tetracyclines and other antibiotics both in the targeted pathogens and in bystander colonizing bacteria that have pathogenic potential. Supporting this concern, daily use of doxycycline selected for increased tetracycline resistance in the skin microbiome [4]. Additionally, the US-based DoxyPEP study found increased rates of *Staphylococcus aureus* doxycycline resistance among participants receiving doxy-PEP, though the results were not statistically significant in the setting of a small sample size [2].

While increasing rates of tetracycline resistance could create challenges in the treatment of many bacterial pathogens [5], resistance in *S aureus* is of particular concern. Doxycycline is a preferred therapy for stepdown and outpatient management of *S aureus*, including methicillin-resistant *S aureus* (MRSA) [6]. Furthermore, because resistance tends to aggregate in certain strains, selection of tetracycline-resistant *S aureus* by doxy-PEP could lead to co-selection for strains that are resistant to other anti-staphylococcal antibiotics [7]. Here, we sought to characterize patterns of *S aureus* resistance and co-resistance in patients who may be prescribed doxy-PEP in an effort to understand the potential impact of widespread doxy-PEP adoption. We hypothesized that tetracycline-intermediate/resistant isolates were more likely than tetracycline-susceptible isolates to be co-resistant to other important anti-staphylococcal antibiotics.

## METHODS

We performed a retrospective analysis of all *S aureus* cultures in people with a sex of male reported in the electronic medical record (EMR) who either had a diagnosis of human immunodeficiency virus (HIV) or were prescribed HIV preexposure prophylaxis (PrEP), were 18 years of age or older, and were receiving care at 2 academic medical centers in Boston, Massachusetts, between June 2015 and May 2022. Patients were identified using a clinical data repository containing microbiologic data, diagnosis codes, and medication prescriptions from the EMR. Because the number and gender of sexual partners was not reliably recorded in the EMR, HIV positivity and receipt of HIV PrEP were used to identify patients whose sexual behaviors were most likely to qualify them for doxy-PEP under current guidelines [3]. We assessed the frequency of bacterial STIs in our study cohort by searching for positive gonorrhea/chlamydia nucleic acid amplification tests (NAATs) and positive (≥1:1) rapid plasma reagin (RPR) tests that had been performed between June 2015 and May 2022.

Notably, people who inject drugs have higher rates of both HIV and *S aureus* infections but are not currently considered eligible for doxy-PEP. There is not an *International Classification of Diseases, Tenth Revision* diagnosis code specifically associated with intravenous drug use, but an estimated 75% of people who inject drugs in North America primarily inject opioids [8]. In an effort to exclude people who inject drugs, we removed all patients with a diagnosis code containing the word “opioid” from our study cohort. A similar strategy has been used to identify patients with endocarditis attributable to injection drug use [9].

We sought to de-duplicate isolates to prevent individuals who were sampled repeatedly from skewing estimates of antimicrobial resistance. There are several strategies for de-duplication. The World Health Organization, for example, recommends inclusion of only 1 isolate per patient per specimen type primarily to increase the feasibility of data collection across healthcare systems, particularly those with limited resources [10]. In our analysis, we chose to define unique episodes of infection as all cultures that were positive for *S aureus* with identical antibiotic susceptibility profiles within a 14-day period. Isolates were considered distinct if their susceptibility to any antibiotic was ≥2 two-fold dilutions removed from the minimum inhibitory concentration (MIC) of the first isolated strain. If multiple *S aureus* specimens with distinct susceptibility profiles were isolated during a given 14-day period, all were included. Nasal swabs performed for MRSA surveillance were not included because they were not assessed for tetracycline susceptibility.

Susceptibility of *S aureus* isolates to tetracycline, doxycycline, oxacillin, trimethoprim-sulfamethoxazole (TMP/SMX), and clindamycin were defined by Clinical and Laboratory Standards Institute (CLSI) MIC breakpoints [11]. Isolates with inducible resistance to clindamycin were categorized as resistant. The frequency of resistance to oxacillin, TMP/SMX, and clindamycin in tetracycline-susceptible *S aureus* isolates (MIC ≤4.0 μg/mL) was compared to the frequency of resistance in the same antibiotics among tetracycline-intermediate (MIC = 8 μg/mL) or tetracycline-resistant (MIC ≥16 μg/mL) isolates. Rates of institutional resistance were calculated using institution-specific antibiograms from 2022 that reported the resistance patterns of 5777 total isolates (2300 from the Brigham and Women's Hospital and 3477 from the Massachusetts General Hospital). The antibiograms draw from all specimens submitted to a Mass General Brigham laboratory, which includes both inpatients and outpatients.

Fisher exact test was used to assess resistance co-occurrence and adjusted for multiple comparisons using the Bonferroni correction. Fisher exact test was also used to compare rates of resistance in our study cohort to rates of institutional resistance. Logistic regression was used to assess for association between year of sampling and resistance over the study period. Statistical tests were performed in R software (version 4.1.2, R Core Team 2021) [12], and the *tidyverse* package was used for data analysis and visualization [13].

This study was approved by the Institutional Board (IRB) of Mass General Brigham (IRB number 2003P000336).

## RESULTS

**Figure 1.**
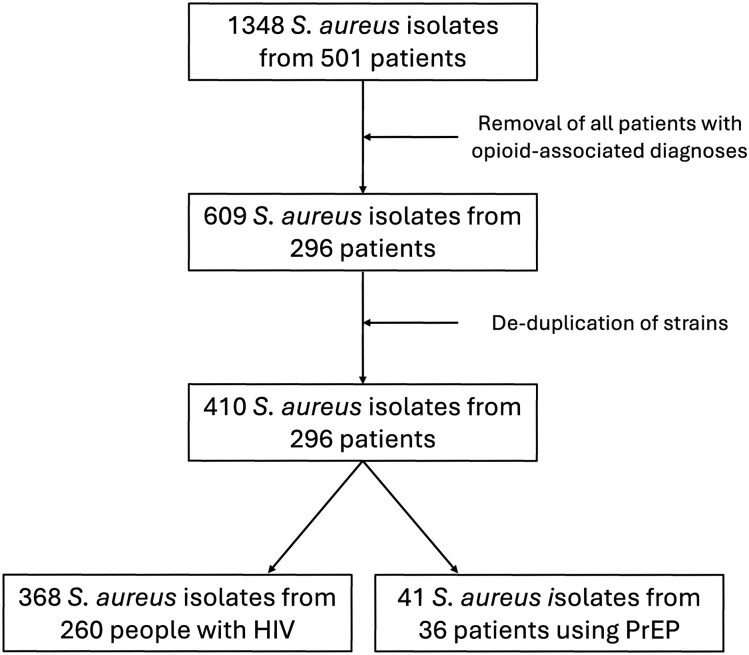
Cohort identification schematic. Abbreviations: HIV, human immunodeficiency virus; PrEP, preexposure prophylaxis.

We identified 410 isolates of *S aureus* collected from 296 patients (Figure 1). Three hundred sixty-nine (90%) *S aureus* isolates were obtained from 260 people with HIV, and 41 (10.0%) *S aureus* isolates were obtained from 36 patients using HIV PrEP. We found that 86 (29.0%) of the patients in our cohort had either a positive gonorrhea/chlamydia NAAT or an RPR with a titer of ≥1:1 during the study period (Supplementary Table 1). People with a history of bacterial STIs contributed 114 (27.8%) of the isolates in our study cohort.

The mean number of isolates collected per patient was 1.36, with a median of 1 and a maximum of 6 (Supplementary Table 2). Most isolates were obtained from a skin/soft tissue source (159 [38.8%]), an abscess (70 [17.1%]), the lungs (61 [14.9%]), or blood (24 [5.9%]) (Supplementary Table 3). While there was no statistically significant association between resistance to doxycycline, clindamycin, TMP/SMX, or oxacillin and the year of sampling, there was a statistically significant association between the year of sampling and resistance to tetracycline (*P* = .02), which increased over the study period (Supplementary Figure 1). Isolates from our patient cohort were significantly more resistant to oxacillin and clindamycin than the institutional averages reported in 2022 (Supplementary Tables 4 and 5).

Of the 410 isolates, 409 (99.8%) had tetracycline susceptibility data, and 294 (71.7%) had doxycycline susceptibility data. Among isolates with tetracycline susceptibility data, 54 (13.2%) isolates were tetracycline resistant (MIC ≥16 μg/mL) and 2 (0.5%) isolates were tetracycline intermediate (MIC = 8 μg/mL). Among isolates with doxycycline susceptibility data, 6 (2.0%) were doxycycline resistant (MIC ≥16 μg/mL) and 7 (2.4%) were doxycycline intermediate (MIC = 8 μg/mL).

Patterns of co-resistance associated with tetracycline nonsusceptibility are summarized in Table 1. There was no statistically significant association between tetracycline nonsusceptibility and oxacillin resistance (odds ratio, 1.58 [95% confidence interval {CI}, .75–3.28]; *P* = .42). However, tetracycline-nonsusceptible isolates were 4.52 times more likely to be resistant to TMP/SMX (95% CI, 1.56–12.42; *P* = .001) and 3.62 times more likely to be resistant to clindamycin (95% CI, 1.71–7.94; *P* < .001) than tetracycline-susceptible isolates. The results were similar among the subset of our study cohort with HIV, with a statistically significant association between tetracycline nonsusceptibility and resistance to TMP/SMX and clindamycin (Supplementary Table 6). We were not able to draw any meaningful conclusions about co-resistance among patients using HIV PrEP in the setting of a small sample size (Supplementary Table 7). In the subset of our study cohort with a history of a bacterial STI, there was a statistically significant association between tetracycline nonsusceptibility and resistance to clindamycin, but not to any other antibiotics (Supplementary Table 8).

**Table 1.** Odds of Resistance to Oxacillin, Trimethoprim-Sulfamethoxazole, and Clindamycin in the Setting of Tetracycline Nonsusceptibility

Resistance	Resistance	Susceptibility	OR (95% CI)	*P* Value
Oxacillin	OXA Resistant	OXA Susceptible		
TET-I/R	27/56 (48.2%)	29/56 (51.7%)	1.58 (.75–3.28)	.42
TET-S	131/353 (37.1%)	222/353 (62.9%)	…	
TMP/SMX	TMP/SMX Resistant	TMP/SMX Susceptible		
TET-I/R	12/56 (21.4%)	44/56 (78.6%)	4.52 (1.56–12.42)	.001
TET-S	20/353 (5.7%)	333/353 (94.3%)	…	
Clindamycin	CLI Resistant	CLI Susceptible		
TET-I/R	36/56 (64.3%)	20/56 (35.7%)	3.62 (1.71–7.94)	<.001
TET-S	117/353 (33.1%)	236/353 (66.9%)	…	

Counts and percentages of *Staphylococcus aureus* isolates that are susceptible to tetracycline (minimum inhibitory concentration [MIC] ≤4.0 μg/mL) or intermediate/resistant to tetracycline (MIC ≥8.0 μg/mL) and susceptible (MIC ≤2.0 μg/mL) or resistant (MIC ≥4.0 μg/mL) to oxacillin, susceptible (MIC ≤2/38 μg/mL) or resistant (MIC ≥4/76) to TMP/SMX, or susceptible (MIC ≤0.5 μg/mL without inducible resistance) or resistant (MIC ≥4 μg/mL or positive for inducible resistance) to clindamycin. ORs of co-resistance to oxacillin, TMP/SMX, and clindamycin in the setting of tetracycline nonsusceptibility are reported. There was a significant association between tetracycline nonsusceptibility and resistance to TMP/SMX and clindamycin.

Abbreviations: CI, confidence interval; CLI, clindamycin; I/R, intermediate/resistant; OR, odds ratio; OXA, oxacillin; S, susceptible; TET, tetracycline; TMP/SMX, trimethoprim-sulfamethoxazole.

We assessed rates of resistance to doxycycline in tetracycline-susceptible and tetracycline-intermediate/resistant isolates. Of 294 isolates with doxycycline susceptibility data, 257 (87.4%) were tetracycline susceptible and 37 (12.6%) were tetracycline nonsusceptible. Among tetracycline-susceptible isolates, 257 (100%) were also susceptible to doxycycline. Among tetracycline-nonsusceptible isolates (MIC ≥8.0 μg/mL), 24 (64.9%) were doxycycline susceptible, 7 (18.9%) were doxycycline intermediate, and 6 (16.2%) were doxycycline resistant **(**Supplementary Figure 2).

## DISCUSSION

Frequent unprotected sexual encounters have been associated with community-acquired MRSA in MSM with HIV [14]. In keeping with this finding, *S aureus* infections in our cohort of doxy-PEP–eligible patients demonstrated increased rates of resistance to commonly used anti-staphylococcal antibiotics when compared to the institutional average. Furthermore, we found that tetracycline nonsusceptibility in *S aureus* was significantly associated with co-resistance to 2 anti-staphylococcal antibiotics, TMP/SMX and clindamycin, in a cohort of patients with a sex of male reported in the EMR who were either HIV positive or prescribed HIV PrEP. While doxy-PEP offers a promising strategy to decrease the frequency of bacterial STIs, increased selective pressure from higher rates of doxycycline use [15] could foster tetracycline resistance in *S aureus* and other common pathogens via bystander selection. Our results suggest that in selecting for tetracycline resistance, doxy-PEP could select for strains of *S aureus* that are also resistant to TMP/SMX and clindamycin.

Limitations to this study include using HIV positivity and PrEP as a proxy for identifying those who might be prescribed doxy-PEP. This strategy likely excluded some who might be doxy-PEP eligible, such as MSM and TGW who are HIV negative and not on PrEP, and included others in whom doxy-PEP is not currently recommended, such as men who have sex with women. Because the majority of patients included in this study (260 [87.8%]) were living with HIV, these results may not generalize to people who are HIV negative. Finally, the Centers for Disease Control and Prevention gives the strongest recommendation for doxy-PEP use in people with a history of bacterial STIs in the past 12 months. Only 86 (29.0%) of the patients in our study cohort had laboratory evidence of a bacterial STI over the study period. Among this group, there was a significant association between tetracycline resistance and clindamycin resistance, but not between tetracycline resistance and TMP/SMX resistance.

Notably, we used tetracycline susceptibility as a proxy for susceptibility to doxycycline, following CLSI guidelines [9]. However, many tetracycline-resistant isolates were reported as doxycycline susceptible. Our cohort included too few examples of isolates with doxycycline MICs ≥8 µg/mL to draw meaningful conclusions about co-inheritance of resistance to other antibiotics at this threshold. Neither the tetracycline nor the doxycycline MICs that would offer *S aureus* a selective advantage in the context of doxy-PEP, which is dosed differently than traditional courses of antibiotics, are known at this time. Given these unknowns and the limitations of our dataset, we made the conservative choice to evaluate at the CLSI cutoff for tetracycline nonsusceptibility.

To date, studies examining development of antimicrobial resistance in *S aureus* after use of doxy-PEP have been limited in size and focused on resistance to doxycycline. Our findings suggest that the doxy-PEP–eligible population is already more likely to carry resistant strains of *S aureus.* Furthermore, use of doxy-PEP in this population may select for strains of *S aureus* that carry resistance not only to tetracyclines, but also to other common anti-staphylococcal antibiotics. The extent to which doxy-PEP will select for these strains and the efficiency with which resistant strains will be transmitted is unknown. This work underlines the importance of monitoring *S aureus* resistance to tetracyclines and to other classes of antibiotics in patients taking doxy-PEP.

## Supplementary Material

jiae634_Supplementary_Data

## Supplementary Data

Supplementary materials are available at *The Journal of Infectious Diseases* online (http://jid.oxfordjournals.org/). Supplementary materials consist of data provided by the author that are published to benefit the reader. The posted materials are not copyedited. The contents of all supplementary data are the sole responsibility of the authors. Questions or messages regarding errors should be addressed to the author.

## Notes

***Acknowledgments.*** The authors thank Kirstin Roster and Thi Mui Pham for helpful comments on the manuscript.

***Author contributions.*** Y. H. G. and R. M. designed this study, and Y. H. G. supervised this work. R. M. acquired patient data with guidance from G. K. R. and culture data with assistance from S. K., and D. H. provided guidance on data management and analysis in R software. R. M. and Y. H. G. wrote the manuscript. All authors reviewed the manuscript for scientific content and approved the final version.

***Data and code availability.*** Analysis codes are available at https://github.com/rmittelstaedt/Doxy_PEP_and_S_aureus_Resistance.

***Financial support*.** This work was supported by the National Institute of Allergy and Infectious Diseases (grant number T32 AI007433). This work was also funded in part by contract number 200-2016-91779 with the Centers for Disease Control and Prevention.
