# Supplementary material for: Staphylococcus aureus Tetracycline Resistance and Co-resistance in a Doxycycline Postexposure Prophylaxis–Eligible Population
Source: J Infect Dis. 2024 Dec 24;231(4):e708–12. doi: 10.1093/infdis/jiae634 (PMC11998573; doi:10.1093/infdis/jiae634)
Supplement: jiae634_Supplementary_Data [file jiae634_supplementary_data.zip › 1_6_25_Supplement_Final_Clean.docx]

**Supplemental Material**

**Figure S1**: **Resistance Trends in *S. aureus* from 2015 – 2022**

The percentage of *S. aureus* isolates in our cohort resistant to clindamycin, doxycycline, oxacillin, tetracycline, and TMP/SMX between June 1, 2015, and May 2, 2022. TMP/SMX = trimethoprim-sulfamethoxazole. This figure was prepared in R (version 4.1.2, R Core Team 2021)^1^ using the *tidyverse*^2^ and *jtools*^3^ packages.

**Figure S2: Correlation of Tetracycline Resistance to Doxycycline Resistance**

**A.** Counts and percentages of *S. aureus* isolates that are susceptible to tetracycline (TET-S, MIC ≤ 4.0 μg/mL) or intermediate/resistant to tetracycline (TET-IR, MIC ≥ 8.0 μg/mL) and susceptible (MIC ≤ 4.0 μg/mL), intermediate (MIC = 8), or resistant (MIC ≥ 16 μg/mL) to doxycycline. **B.** A magnified view of counts and percentages of tetracycline intermediate/resistant isolates by doxycycline minimum inhibitory concentrations. All isolates described in Panel B have a tetracycline MIC ≥ 8.0 μg/mL. Only 35.1% of tetracycline non-susceptible isolates were also doxycycline non-susceptible. This figure was prepared in R (version 4.1.2, R Core Team 2021)^1^ using the *tidyverse*^2^ package.

**Table S1: Patients with a History of Bacterial Sexually Transmitted Infections**

The number of patients in our cohort who were positive for *N. gonorrhoeae* or *C. trachomatis* by nucleic acid amplification testing or had a positive reactive plasma reagin test indicating a prior history of syphilis during the study period. We found that 86 unique patients (29.1% of our cohort) had a history of 114 bacterial sexually transmitted infections.

| **Sexually Transmitted Infection** | **Number of Patients** |
| --- | --- |
| Gonorrhea | 29 |
| Chlamydia | 17 |
| Syphilis | 70 |
| Total Unique Patients | 86 |

**Table S2: Number of Specimens Contributed per Patient**

This table describes the number of isolates contributed per patient. 296 total patients contributed 410 total specimens, with a mean number of 1.36 isolates per patient. Clinical records of patients contributing more than 10 specimens were manually reviewed. In two cases, review indicated patients with 20 specimens had chronic infection or colonization with *S. aureus*, in which the specimens did not reflect distinct episodes of acute infection. In these cases, only unique strains of *S. aureus* were included in the final analysis, reducing the total number of specimens from these patients to 6 each.

| **Number of Specimens Contributed per Patient** | **Number of Patients** |
| --- | --- |
| 1 | 233 |
| 2 | 45 |
| 3 | 14 |
| 4 | 3 |
| 5 | 3 |
| 6 | 3 |

**Table S3: *Staphylococcus aureus* specimen types in the study cohort.**

| **Specimen Type** | **Number (Percentage)** |
| --- | --- |
| Skin/Soft Tissue | 159 (38.8%) |
| Abscess | 70 (17.1%) |
| Pulmonary | 61 (14.9%) |
| Blood | 24 (5.9%) |
| Urine | 19 (4.6%) |
| Bone/Joint | 11 (2.7%) |
| Other | 2 (0.5%) |
| Uncategorized | 64 (15.6%) |
| Total | 410 |

**Resistance in the study cohort relative to 2022 institutional resistance**

Antibiograms at the Brigham and Women’s Hospital and Massachusetts General Hospital are prepared using the isolate de-duplication methodology advised in the CLSI M39 document.^4^ These guidelines recommend capturing only the first isolate from a given patient in a calendar year regardless of strain or body site. As described in the main text, we de-duplicated our isolates using a method intended to capture each unique episode of infection. **Table S4** compares our cohort to the institutional antibiograms when our cohort is de-duplicated via the per-infection method and the institutional antibiograms are de-duplicated via the CLSI method. **Table S5** compares rates of resistance when both our cohort and the institutional antibiograms are de-duplicated via the CLSI method. The CLSI method is more restrictive and identified only 331 unique specimens in our cohort compared to 410 specimens identified by the per-infection method described in the main text. Notably, the vast majority (260/296, 87.8%) of the patients in our cohort were living with HIV, and a minority (36/296, 12.2%) were using HIV PrEP.

**Table S4: Resistance in our study cohort (de-duplicated via the per-infection method) relative to institutional resistance (de-duplicated by the CLSI method)**

| **Antibiotic** | **Resistance in Study Cohort** | **Institutional Resistance** | **OR (95% CI, p-value)** |
| --- | --- | --- | --- |
| **Tetracycline** | 56/409 (13.7%) | 244/2300 (10.6%) | 1.34 (0.96 – 1.84, p = 0.07) |
| **Doxycycline** | 13/294 (4.4%) | 104/3477 (3.0%) | 1.50 (0.76 – 2.72, p = 0.16) |
| **Oxacillin** | 158/409 (38.6%) | 1492/5777 (25.8%) | 1.81 (1.46 – 2.23, p < 0.001) |
| **Trimethoprim-sulfamethoxazole** | 32/409 (7.8%) | 316/5777 (5.5%) | 1.47 (0.97 – 2.15, p = 0.06) |
| **Clindamycin** | 153/409 (37.4%) | 1573/5777 (27.2%) | 1.60 (1.29 – 1.98, p < 0.001) |

**Table S5: Resistance in the study cohort relative to institutional resistance in 2022 when both are de-duplicated using the CLSI method.**

| **Antibiotic** | **Resistance in Study Cohort** | **Institutional Resistance** | **OR (95% CI, p-value)** |
| --- | --- | --- | --- |
| **Tetracycline** | 43/330 (13.0%) | 244/2300 (10.6%) | 1.26 (0.87 – 1.80, p = 0.19) |
| **Doxycycline** | 13/232 (5.6%) | 104/3477 (3.0%) | 1.92 (0.98 – 3.51, p = 0.05) |
| **Oxacillin** | 124/330 (37.6%) | 1492/5777 (25.8%) | 1.73 (1.36 – 2.19, p < 0.001) |
| **Trimethoprim-sulfamethoxazole** | 22/330 (6.7%) | 316/5777 (5.5%) | 1.23 (0.75 – 1.94, p = 0.324) |
| **Clindamycin** | 123/330 (37.3%) | 1573/5777 (27.2%) | 1.59 (1.25 – 2.01, p < 0.001) |

**Table S6: Odds of resistance to oxacillin, trimethoprim-sulfamethoxazole, and clindamycin in the setting of tetracycline non-susceptibility among people living with HIV**

Counts and percentages of *S. aureus* isolates obtained from patients living with HIV that are susceptible to tetracycline (MIC ≤ 4.0 μg/mL) or intermediate/resistant to tetracycline (MIC ≥ 8.0 μg/mL) and susceptible (MIC ≤ 2.0 μg/mL) or resistant (MIC ≥ 4.0 μg/mL) to oxacillin, susceptible (MIC ≤ 2/38 μg/mL) or resistant (MIC ≥ 4/76) to trimethoprim-sulfamethoxazole, or susceptible (MIC ≤ 0.5 μg/mL without inducible resistance) or resistant (MIC ≥ 4 μg/mL or positive for inducible resistance) to clindamycin. Odds ratios (OR) of co-resistance to oxacillin, trimethoprim-sulfamethoxazole, and clindamycin in the setting of tetracycline non-susceptibility are reported. 260 (87.8%) of participants in our study cohort were living with HIV. In line with the results of our main analysis (**Table 1**), there was a significant association between tetracycline non-susceptibility and resistance to TMP/SMX and clindamycin. TET = tetracycline; OXA = oxacillin; TMP/SMX = trimethoprim-sulfamethoxazole; CLI = clindamycin.

| **Oxacillin** | | | |
| --- | --- | --- | --- |
|  | **OXA-Resistant** | **OXA-Sensitive** | **OR (CI, p-value)** |
| **TET-Intermediate/Resistant** | 27 (50.0%) | 27 (50.0%) | 1.64 (0.77 – 3.49, p = 0.30) |
| **TET-Sensitive** | 119 (37.9%) | 196 (62.2%) |  |
| **Trimethoprim-Sulfamethoxazole** | | | |
|  | **TMP/SMX-Resistant** | **TMP/SMX-Sensitive** | **OR (CI, p-value)** |
| **TET-Intermediate/Resistant** | 11 (20.4%) | 43 (79.6%) | 3.96 (1.31 – 11.28, p = 0.004) |
| **TET-Sensitive** | 19 (6.1%) | 296 (94.0%) |  |
| **Clindamycin** | | | |
|  | **CLI-Resistant** | **CLI-Sensitive** | **OR (CI, p-value)** |
| **TET-Intermediate/Resistant** | 34 (63.0%) | 20 (37.0%) | 3.29 (1.54 – 7.32, p < 0.001) |
| **TET-Sensitive** | 107 (34.1%) | 208 (66.0%) |  |

**Table S7: Odds of resistance to oxacillin, trimethoprim-sulfamethoxazole, and clindamycin in the setting of tetracycline non-susceptibility among people taking HIV pre-exposure prophylaxis**

Counts and percentages of *S. aureus* isolates obtained from patients taking HIV pre-exposure prophylaxis that are susceptible to tetracycline (MIC ≤ 4.0 μg/mL) or intermediate/resistant to tetracycline (MIC ≥ 8.0 μg/mL) and susceptible (MIC ≤ 2.0 μg/mL) or resistant (MIC ≥ 4.0 μg/mL) to oxacillin, susceptible (MIC ≤ 2/38 μg/mL) or resistant (MIC ≥ 4/76) to trimethoprim-sulfamethoxazole, or susceptible (MIC ≤ 0.5 μg/mL without inducible resistance) or resistant (MIC ≥ 4 μg/mL or positive for inducible resistance) to clindamycin. Only 36 (12.2%) participants in our study cohort were taking HIV pre-exposure prophylaxis. The sample sizes were too small to draw any conclusions about co-inheritance of resistance in this group. TET = tetracycline; OXA = oxacillin; TMP/SMX = trimethoprim-sulfamethoxazole; CLI = clindamycin.

| **Oxacillin** | | | |
| --- | --- | --- | --- |
|  | **OXA-Resistant** | **OXA-Sensitive** | **OR (CI, p-value)** |
| **TET-Intermediate/Resistant** | 0 (0%) | 2 (100%) | - |
| **TET-Sensitive** | 12 (31.6%) | 26 (68.4%) |  |
| **Trimethoprim-Sulfamethoxazole** | | | |
|  | **TMP/SMX-Resistant** | **TMP/SMX-Sensitive** | **OR (CI, p-value)** |
| **TET-Intermediate/Resistant** | 1 (50.0%) | 1 (50.0%) | - |
| **TET-Sensitive** | 1 (2.6%) | 37 (97.4%) |  |
| **Clindamycin** | | | |
|  | **CLI-Resistant** | **CLI-Sensitive** | **OR (CI, p-value)** |
| **TET-Intermediate/Resistant** | 2 (100%) | 0 (0%) | - |
| **TET-Sensitive** | 10 (26.3%) | 28 (73.7%) |  |

**Table S8: Odds of resistance to oxacillin, trimethoprim-sulfamethoxazole, and clindamycin in the setting of tetracycline non-susceptibility among patients with a history of bacterial sexually transmitted infections**

Counts and percentages of *S. aureus* isolates obtained from people with a history of bacterial sexually transmitted infections that are susceptible to tetracycline (MIC ≤ 4.0 μg/mL) or intermediate/resistant to tetracycline (MIC ≥ 8.0 μg/mL) and susceptible (MIC ≤ 2.0 μg/mL) or resistant (MIC ≥ 4.0 μg/mL) to oxacillin, susceptible (MIC ≤ 2/38 μg/mL) or resistant (MIC ≥ 4/76) to trimethoprim-sulfamethoxazole, or susceptible (MIC ≤ 0.5 μg/mL without inducible resistance) or resistant (MIC ≥ 4 μg/mL or positive for inducible resistance) to clindamycin. Odds ratios (OR) of co-resistance to oxacillin, trimethoprim-sulfamethoxazole, and clindamycin in the setting of tetracycline non-susceptibility are reported. 86 (29.1%) of participants in our study cohort had a history of a bacterial sexually transmitted infection. Only 114 (27.8%) isolates were contributed by these patients, reducing statistical power. In this group, there was a significant association between tetracycline non-susceptibility and resistance to clindamycin. TET = tetracycline; OXA = oxacillin; TMP/SMX = trimethoprim-sulfamethoxazole; CLI = clindamycin.

| **Oxacillin** | | | |
| --- | --- | --- | --- |
|  | **OXA-Resistant** | **OXA-Sensitive** | **OR (CI, p-value)** |
| **TET-Intermediate/Resistant** | 7 (33.3%) | 14 (66.7%) | 0.54 (0.13 – 1.94, p = 0.42) |
| **TET-Sensitive** | 45 (48.4%) | 48 (51.6%) |  |
| **Trimethoprim-Sulfamethoxazole** | | | |
|  | **TMP/SMX-Resistant** | **TMP/SMX-Sensitive** | **OR (CI, p-value)** |
| **TET-Intermediate/Resistant** | 4 (19.0%) | 17 (81.0%) | 3.36 (0.44 – 21.74, p = 0.26) |
| **TET-Sensitive** | 6 (6.5%) | 87 (93.5%) |  |
| **Clindamycin** | | | |
|  | **CLI-Resistant** | **CLI-Sensitive** | **OR (CI, p-value)** |
| **TET-Intermediate/Resistant** | 16 (76.2%) | 5 (23.8%) | 8.51 (2.13 – 44.70, p < 0.001) |
| **TET-Sensitive** | 25 (26.9%) | 68 (73.1%) |  |

**References:**

1. R Core Team. R: A language and environment for statistical computing. R Foundation for Statistical Computing; 2021. Available from: <https://www.R-project.org>

2. Wickham H, Averick M, Bryan J, Chang W, McGowan LD, François R, Grolemund G, Hayes A, Henry L, Hester J, Kuhn M, Pedersen TL, Miller E, Bache SM, Müller K, Ooms J, Robinson D, Seidel DP, Spinu V, Takahashi K, Vaughan D, Wilke C, Woo K, Yutani H. Welcome to the tidyverse. *Journal of Open Source Software*. 2019;4(43):1686. doi: 10.21105/joss.01686.

3. Long JA (2022). jtools: Analysis and Presentation of Social Scientific Data. R package version 2.2.0. Available from: https://cran.r-project.org/package=jtools

4. CLSI. Analysis and Presentation of Cumulative Antimicrobial Susceptibility Test Data; Approved Guideline -- Fourth Edition. CLSI document M39-A4. Wayne, PA. Clinical and Laboratory Standards Institute, 2014.
