# Supplementary figures and images for: Staphylococcus aureus Tetracycline Resistance and Co-resistance in a Doxycycline Postexposure Prophylaxis–Eligible Population
Source: J Infect Dis. 2024 Dec 24;231(4):e708–12. doi: 10.1093/infdis/jiae634 (PMC11998573; doi:10.1093/infdis/jiae634)

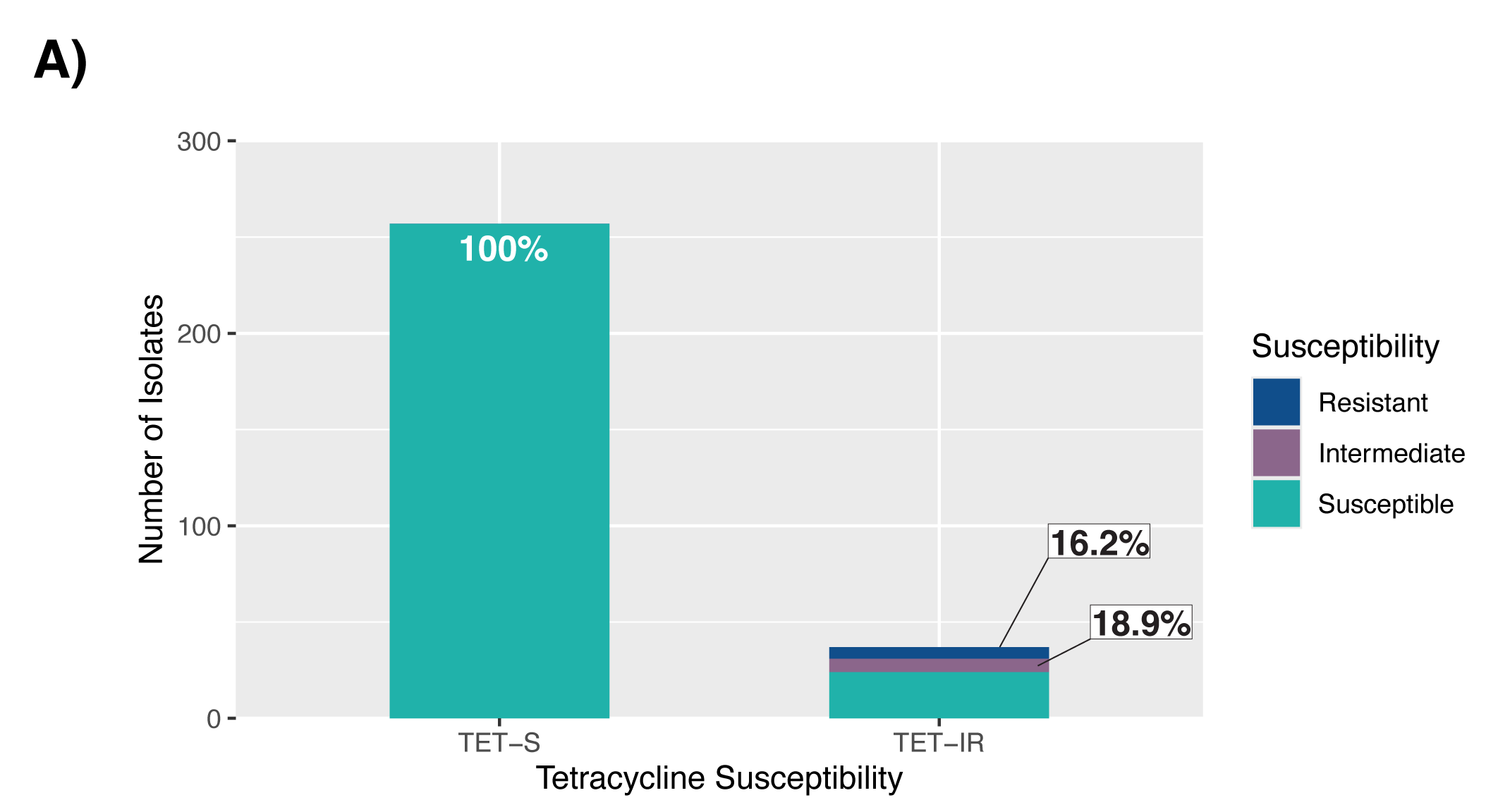

Supplement: jiae634_Supplementary_Data [file jiae634_supplementary_data.zip › suppfig2a_icr_edited.tif]

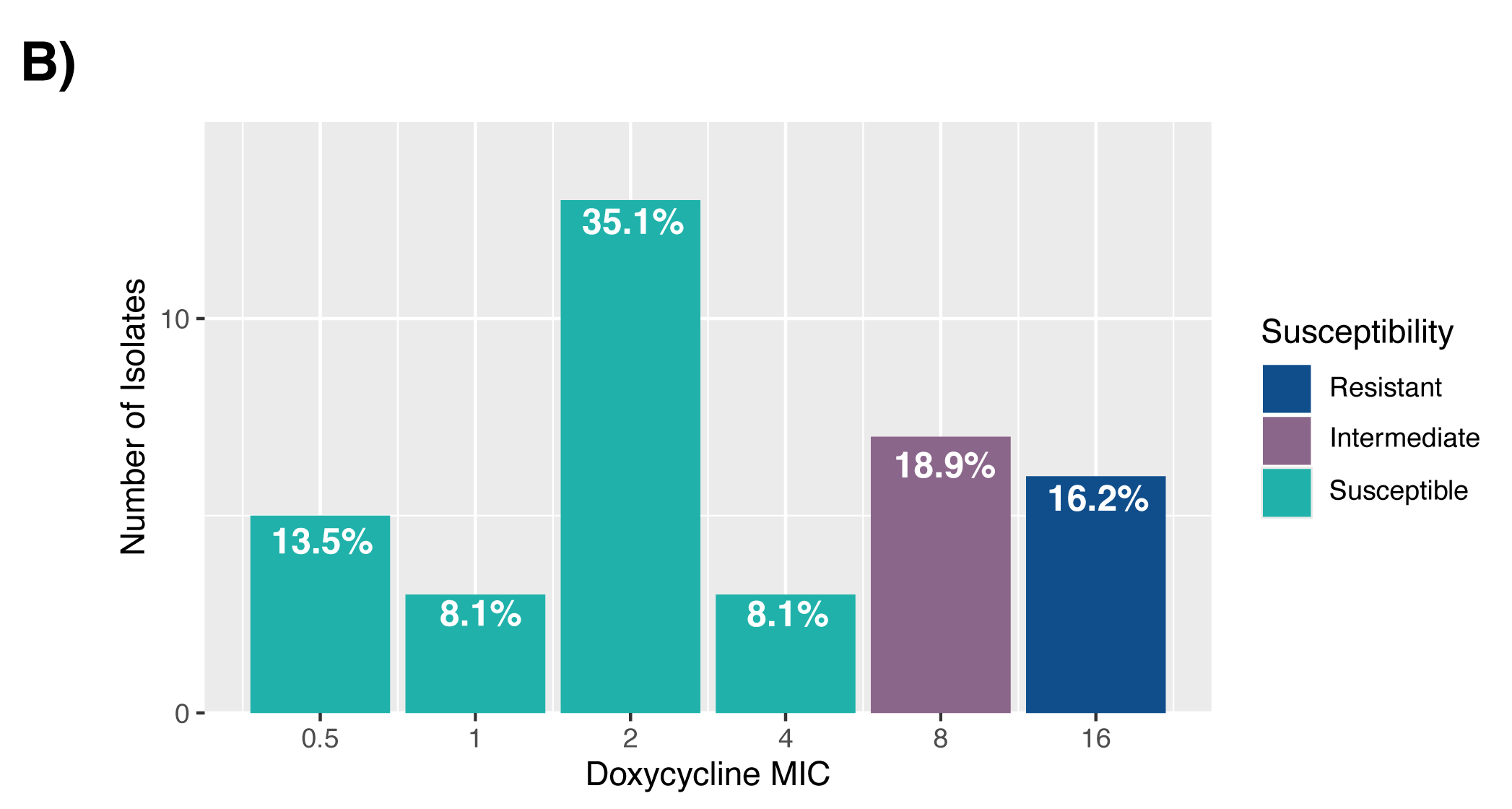

Supplement: jiae634_Supplementary_Data [file jiae634_supplementary_data.zip › suppfig2b_edited.tif]

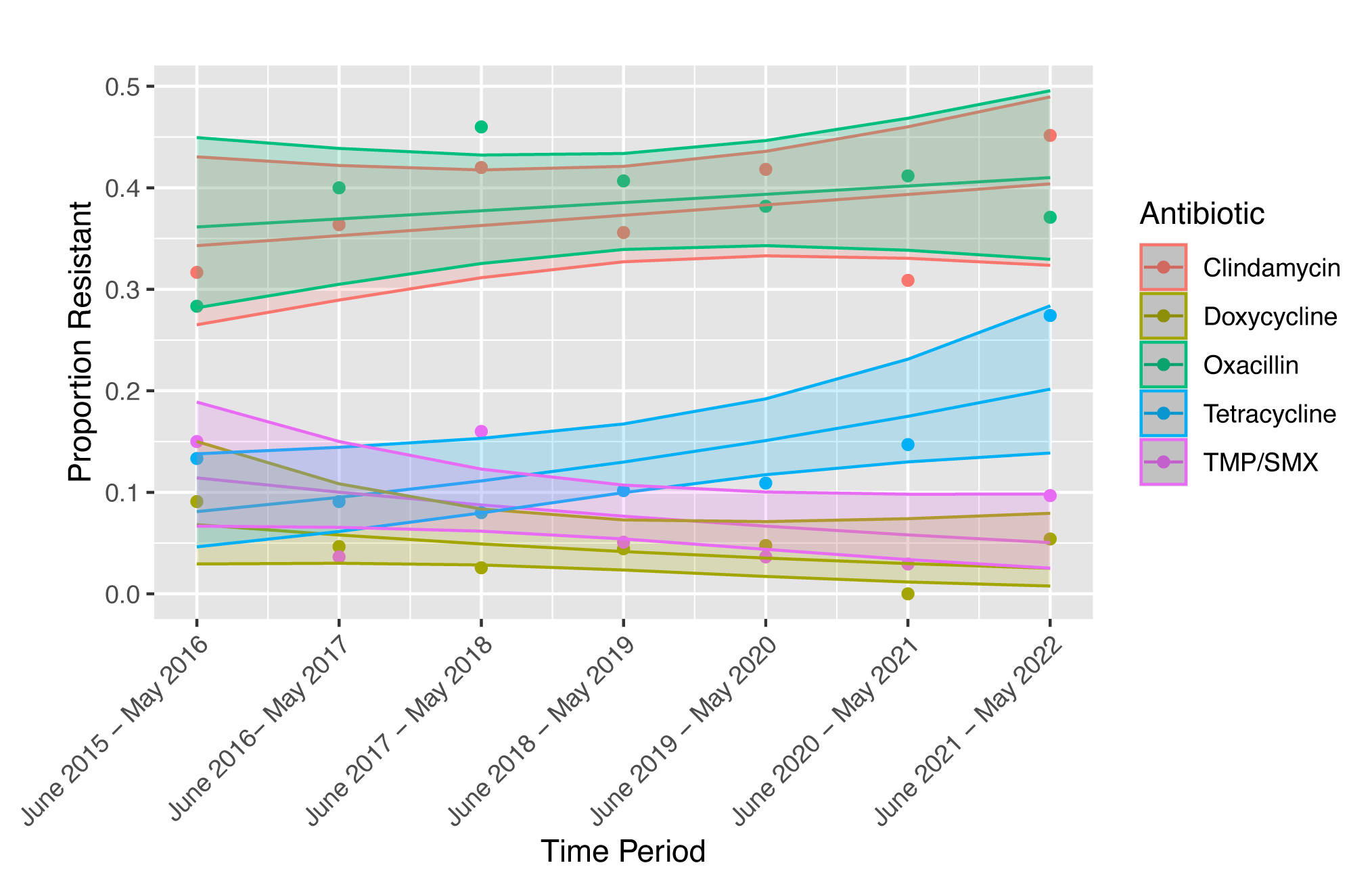

Supplement: jiae634_Supplementary_Data [file jiae634_supplementary_data.zip › suppfig_1_edited.tif]
